# Preterm Prelabour Rupture of Membrane (PPROM) in a Young Female in South-South Nigeria: A Clinical Case Report

**DOI:** 10.7759/cureus.51649

**Published:** 2024-01-04

**Authors:** Queen L Ekpa, Mfonabasi I Udoudo, Emmanuella I Nwebeh, Obinna C Nwebeh

**Affiliations:** 1 General Practice, Conestoga College, Kitchener, CAN; 2 Obstetrics and Gynaecology, University of Uyo Teaching Hospital, Uyo, NGA; 3 Family Medicine, Alex Ekwueme Federal University, Abakaliki, NGA; 4 Family Medicine, Alex Ekwueme Federal University Teaching Hospital, Abakaliki, NGA

**Keywords:** case report, prelabor, underserved community, rupture, membranes, gestational age, premature, preterm

## Abstract

A 30-year-old in a second pregnancy presented with new-onset spontaneous vaginal discharge of clear liquid for two hours at a gestational age of 29 weeks + 6 days; no other symptoms were present. Movement aggravated vaginal fluid leakage. She appeared anxious but otherwise vitally stable. An immediate ultrasound scan revealed reduced liquor volume. Conservative management was followed by the surgical delivery of a live preterm neonate. The neonate was admitted to the Special Care Baby Unit and the mother was monitored post-surgically in the obstetric ward. Several studies have described the etiology, pathogenesis, and various approaches to the management of Preterm Prelabour Rupture of Membranes (PPROM); however, there is no single global guideline for managing this condition. There is a consensus that PPROM is a notable risk factor for preterm labor, and significantly impacts both maternal and neonatal morbidity, as well as neonatal mortality. We compared epidemiology across other countries to the statistics obtained from Nigeria as well as management guidelines. Studies have also described the advantages of conservative management over proactive surgical intervention in improving neonatal outcomes. Moreover, the management of PPROM is affected by healthcare resources in different countries and their national protocol, and the impact is significant in developing countries like Nigeria such as the age of fetal viability and the lack of a national protocol.

This paper explores PPROM and an* *uncommon presentation of spontaneous PPROM in the South-South region of Nigeria with only drainage of liquor. This case exemplifies the management approaches of PPROM in an underserved community and the factors that affect the survival of neonates in these communities. The index patient was initially managed conservatively and subsequently delivered a live preterm neonate surgically with good APGAR (appearance, pulse, grimace, activity, and respiration) scores. We expect this case report to prompt a unifying guideline for managing PPROM cases while encouraging advanced research and financial support of PPROM screening and treatment in low-income countries.

## Introduction

Prelabor rupture of membranes (PROM) is the spontaneous separation of the gestational membranes before labor begins. PROM is not always pathological, as it may be succeeded by labor contractions in a term pregnancy [1]. PROM complicates about 8% of all pregnancies at term, contributing to the global burden of maternal and child morbidity and mortality [2]. When this event occurs before term (37 weeks), it is described as preterm prelabour rupture of membranes (PPROM) [3]. PPROM occurs in about 1% of all deliveries and is twice as common in all African Americans [3]. When PPROM occurs between 16+0 to 22+6 weeks gestation, it is classified as extremely preterm prelabour rupture of membrane (EPPROM) [4]. The age of viability in Nigeria is 28 weeks, with at least a 50% chance of survival [5]. Pregnancies delivered before 27 completed weeks are considered to be periviable [5]. Outcomes of PPROM rely hugely on gestation age (GA) at delivery [3].

Objective

This case report discusses the presentation, management, challenges, and outcomes of spontaneous PPROM in a healthy multigravida, in an underserved population. We selected this case to highlight existing gaps in management guidelines in PPROM. This study 1) identifies the possible causes of PPROM in this population, 2) describes an example of PPROM presentation in underserved communities, 3) reviews the management of the index case and compares current literature and guidelines, and 4) discusses the immediate maternal and neonatal outcomes in the index case.

Etiology and Pathophysiology

Though the specific etiology of PPROM is largely unknown [1], the pathogenesis involves an interplay of multifactorial elements between maternal and neonatal systems. The multifactorial cause of PPROM results in increased intrauterine pressure following an imbalance between uterine collagenase and protease activities [3]. The uterine biochemical environment is influenced by factors such as pressure changes, membrane thickness, and contractions [1] leading to the rupture of fetal membranes [6]. The fetal membranes are made of two closely adherent structures: the amnion and the chorion. These structures consist of diverse epithelial, mesenchymal, and trophoblastic cells, which are all embedded in a collagenous matrix. The amnion is relatively smaller, being only one-fifth of the fetal membrane in size. However, it strongly controls the mechanical response of the fetal membrane [6] and acts as a structural barrier [1]. The chorion is thicker, larger, and more cellular. Its function is mainly immunological, preventing degradation of the amnion and buffering the fetus from the maternal immune system [1].

In normal pregnancies, the integrity of these membranes is maintained till term, after which it ruptures either spontaneously or artificially to signify the onset of labor [6]. Spontaneous rupture of membranes (SROM) at term follows biochemically mediated weakening of fetal membranes, precipitated by stretch forces. This leads to remodeling of the cellular matrix and apoptosis of fetal membrane cells. However, in PPROM, pre-weakening of the fetal membranes can occur from a defective collagen matrix and inflammatory processes that lead to cytokine release and a cascade degradation of the matrix. These inflammatory processes include maternal infections and antepartum hemorrhage such as decidua bleeding and placenta abruption [6].

The chorion-amnion interface is less hydrophobic in studied PPROM cases [1] but is usually significantly hydrophobic and frictionless in normal pregnancies. The reduced hydrophobic state implicated in PPROM has been suggested to cause increased friction between the two fetal membranes. Moreover, the region of fetal membranes overlying the maternal cervix has a decreased connective tissue thickness and a decreased rupture strength, thus it is known as the physiologic weak zone in the membrane, also seen in normal pregnancies [1,6]. Rupture is suggested to begin in this zone, leading to the pre-weakening of the membranes, however, in PPROM, it is usually mediated by inflammatory factors. Conditions such as polyhydramnios and twin gestation are associated with increased stretching of fetal membranes [5] while oligohydramnios, BMI < 18.5 kg/m^2^, vitamin deficiency, and cigarette smoking are also identified risk factors for PPROM [1,7].

Cervical incompetence has also been implicated in the pathophysiology of PPROM by causing fetal sac exposure to mechanical forces [6]. This is because the mechanical forces are concentrated on a small surface area, and the effect on the protruding membranes is also higher. The exposed sac also has a higher chance of infection, which will activate the inflammatory cascade and lead to PPROM. A combination of these mechanisms is connected to PPROM pathogenesis. The presence of risk factors such as infection, maternal or fetal stress, excessive uterine stretch, and uterine bleeding will disrupt uterine quiescence, activate the mechanisms, and lead to PPROM [6].

## Case presentation

The index patient is a 30-year-old gravid 2 Para 1 Alive 1 (G2 P1 A1) woman, now P2 A2, with spontaneous drainage of fluid per vagina about two hours before presentation. The fluid was copious and aggravated by the slightest movement. She had no fever, abnormal vaginal discharge or bleeding, or lower urinary tract symptoms. No contractions or labor pains before or during the onset of symptoms. The patient’s last menstrual period (LMP) was the 3rd of February 2022, and the estimated date of delivery (EDD) was the 10th of November, 2022. The estimated gestational age (GA) at presentation was 29 weeks + 6 days from LMP. She had a regular menstrual cycle occurring every 28 days with 3 to 4 days of normal flow. There was no history of hypertension or diabetes in the patient prior to conception or during pregnancy. There is no history of abortions, preterm delivery, or instrumental delivery. There was also no history of cervical trauma from previous vaginal delivery or postpartum hemorrhage. She was regular with her routine antenatal visits. She does not take alcohol or tobacco in any form. There was no family history of preterm deliveries, hypertension, and diabetes.

Physical examination revealed an anxious patient, in no obvious respiratory or painful distress, afebrile with a temperature of 36.2°C, not icteric, not pale or cyanosed, and nil digital clubbing or pedal edema. She had no signs of dehydration, and her vitals were stable. Abdominal examination showed a symphysiofundal height of 31 cm, and a fetus in transverse lie. A fetal heart check with sonic aid showed the presence of a fetal heart rate of 130 beats per minute. There were no visible or palpable uterine contractions. A vaginal examination with a sterile speculum showed clear amniotic fluid pooling in the posterior fornix of the vagina. With the Valvasa maneuver, there was leakage from an open cervix, about two centimeters dilated. The patient was admitted and management began with a provisional diagnosis of PPROM. She was commenced on intravenous fluids, antibiotics (per oral erythromycin 500 mg 6-hourly for 24 hours), salbutamol 4 mg three times daily for 48 hours, and steroids (i.e., dexamethasone 12 mg 12-hourly intramuscularly for 24 hours). Investigations were carried out, as shown in Table 1. Table 2 summarizes fetal parameters from a scan done at a previous visit.

**Table 1:** Results of laboratory and radiological investigations at presentation and diagnosis of PPROM PCV = packed cell volume; HIV = human immunodeficiency virus; HBV = Hepatitis B virus; HCV = Hepatitis C virus; VDRL = venereal disease research lab; US = ultrasound; GA = gestational age; EFW = estimated fetal weight; AFI = amniotic fluid index

Investigations	Results
Full blood count	PCV = 32% (normal: 36 – 46%)
	Hemoglobin = 11.1g/dl (normal: 12 -16 g/dl)
	Total white cell count = 9.3 x 10^9^/L (normal: 3.0 – 13.0 x 10^9^/L)
Retroviral screening	HIV = Negative
	HBV = Negative
	HCV = Negative
	VDRL = Negative
Urinalysis screen	Normal
Urgent Bedside Ultrasound scan	Viable fetus with reduced liquor volume-one pocket of fluid seen (normal: four pockets of fluid)
	US GA = 29 weeks + 4 days
	US EFW = 1.3 kg
	AFI = 0.8 cm (normal: 5-25 cm)
	Placenta location = anterofundal

**Table 2:** Fetal parameters from the result of an obstetric scan done at a previous prenatal visit at 23 weeks gestation BPD = biparietal diameter; HC = head circumference; FL = femur length; AC = abdominal circumference; EDD = estimated date of delivery; FHR = fetal heart rate

Fetal Parameters	Result/ Value
BPD	Measured 65 mm (corresponds to 22 weeks 6 days)
HC	Measured 233 mm (corresponds to 23 weeks 2 days)
FL	Measured 38 mm (corresponds to 23 weeks 2 days)
AC	Measured 195 mm (corresponds to 23 weeks 1 day)
EDD	03/11/2022 ± 2 weeks
Sex	XX
FHR	148 bpm
Liquor volume	Adequate

The report of an ultrasound scan done at a previous visit at 22 weeks + 2 days revealed a normal second-trimester cyesis. The uterus containing a singleton viable fetus in cephalic presentation and a longitudinal lie was visualized. The placenta was normal, and the placenta was anterior. No fetal abnormality was noted, and the cervix was closed. The estimated fetal weight was 690 g noted to be normal for the age. Fetal parameters, which corresponded to GA of 23 weeks 2 days ± 2 weeks, are summarized in Table 2 above.

The patient was placed on bed rest for three days under close monitoring. She delivered a live female neonate with a birthweight of 1.4 kg by emergency lower segment cesarean section after 72 hours of conservative management and no improvement in presenting symptoms. Indications for surgical delivery included severe oligohydramnios secondary to PPROM, and transverse fetal lie. Neonatal APGAR scores were 9/10 and 10/10 in the first and fifth minutes respectively. The neonate was transferred to a Special Care Baby Unit (SCBU) of a Pediatric Specialist Hospital for expert care. The patient's and her baby's care required a multidisciplinary team of the obstetrician, midwife, nursing staff, pediatrician, anesthesiologist, the general physician who admitted the patient, and other supporting staff.

Initially, the patient recuperated very well postoperatively but subsequently developed a fever (which was low-grade, intermittent, and relieved by paracetamol injections), body pains, and chest pains four days postop. There was no undue abdominal pain, lower urinary symptoms, or abnormal vaginal discharge. Chest examination showed coarse crepitation in both lung bases. Abdominal examination showed a clean and dry op-site and well-apposed edges. Lochia was normal on vaginal examination. A diagnosis of pneumonia supported by chest X-ray findings of patchy, bilateral opacities was made, and the patient was treated with antibiotics (i.e., amoxicillin/clavulanic acid 1.2 g intravenously 12 hourly for three days). She continued on oral cefixime 200 mg twice daily for another seven days. The patient was discharged seven days postop, and her baby was discharged after two weeks of intensive care at the SCBU at 1.6 kg. She presented with her baby at two weeks and six weeks post-discharge for follow-up visits, which were satisfactory. The baby continued pediatric specialist follow-up visits and has currently achieved age-appropriate milestones at one year of age.

## Discussion

Literature review and comparison of epidemiological studies

**Figure 1:**
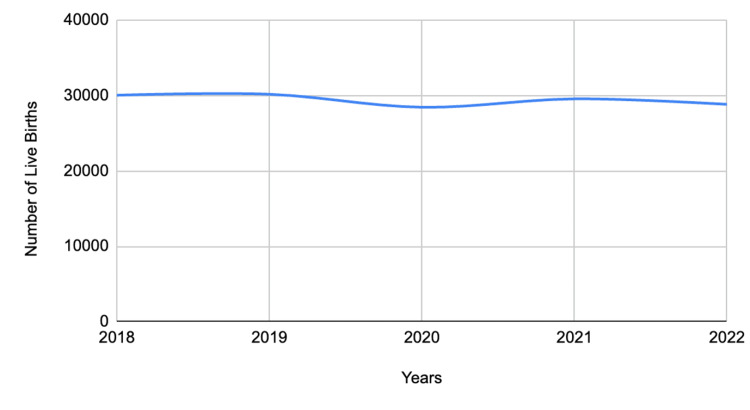
Trends in live preterm gestation (< 37 weeks) in Canada for both sexes between 2018 and 2022 Data from Statistics Canada [11]

In the United States and Nigeria, the incidence of PPROM is 1.4% and 3.3%, respectively [8], whereas the incidence of PROM cases in general in Nigeria is 4.2% [9]. A surveillance period in the United Kingdom (UK) between 1st September 2019 and 28th February 2021 revealed that the exact incidence of EPPROM is unknown and that there are no clear counseling and management guidelines for EPPROM [4]. In 2018, the incidence of PROM in Canada was 8% and incurred healthcare costs of $8 billion per year [10], and by 2022, there were 28,839 preterm live births out of a total of 351,679 total live births [11]. In Ontario, Canada, 3.3% of women had PROM as compared with the Ontario-wide rate of 1.6% [12]. The prevalence of PPROM in Ethiopia, Uganda, Egypt, and China is reported to be 13.7%, 7.5%, 5.3%, and 2.7%, respectively [8]. There is insufficient data in Nigeria and Africa on the outcomes of the treatment of PPROM as well as healthcare expenditure incurred in managing PROM cases [9]. The index case had a PPROM and was otherwise a healthy multigravida woman with an uncomplicated prenatal period and unremarkable medical and antenatal history. Figure 1 below summarizes trends in live preterm births in Canada within four years.

A clinical trial in the US and Spain used cervical assessment by transvaginal ultrasound (TVU) to measure cervical length. Cervical length screening was used to prevent preterm delivery in pregnancies at 14-32 weeks GA [13]. Women who had undergone prior preventive interventions (progesterone, cerclage) for short cervical length were excluded. The general results were inconclusive with low-quality evidence. Thus, there is limited data to suggest that screening with TVU-measured cervical length prolongs pregnancies or prevents PPROM. This requires a clear protocol for the management of women based on measured TVU [13]. The index patient did not have a cervical length screening for PPROM, despite a previous history of vaginal birth, as it is not routinely done in the region.

In a retrospective Romanian study between October 2015 to November 2020, 3% of pregnancies between 24 + 0/7 and 36 + 6/7 gestation were complicated by PPROM, with a worse prognosis in premature infants compared to term infants [7]. This study showed a strong correlation between expectant management of PPROM and neonatal outcomes, thus, demonstrating the advantage of expectant management of PPROM over immediate birth within 48 hours of diagnosis. The limitation of the study was that the exact time between the ROM and presentation at the emergency could not be ascertained, as well as the lack of standard protocol and antibiotic resistance [7]. The index case was placed on bed rest for three days, following which operative cesarean delivery was done. This increased the risk of infections, as there was a lack of resources and patient financial constraints for long-term conservative treatment. Moreover, a transverse fetal lie as in this case, further reduced the likelihood of a vaginal delivery whether induced or spontaneous. However, prophylactic antibiotics and steroids enhanced maternal and neonatal outcomes.

A Chinese study revealed the incidence of PROM at three Chinese hospitals to be 18.72% [2]. The study showed that the rate of PROM varied for different gestation weeks and implicated *Candida* *albicans* colonization as the most frequent bacteria detected from vaginal swabs, with *Escherichia coli* and Group B Streptococcus being the second and third most frequent respectively. For term neonates, PROM was seen to be a risk factor for neonatal infectious diseases, early-onset pneumonia, and neonatal early-onset sepsis. It also showed that clinical chorioamnionitis was associated with a higher risk of neonatal infectious diseases and early-onset sepsis [2]. Cesarean section was further identified as a risk factor for early-onset neonatal pneumonia. Multiparity was a protective factor of infectious diseases possibly due to the shorter time of duration of labour [2]. A major drawback of this study was that the number of early-onset sepsis cases was not sufficient to assess the influence of maternal usage of antibiotics. The index patient did not have microbial swabs taken for culture to rule out asymptomatic chorioamnionitis as a risk factor, as her history did not suggest a genital tract infection.

**Figure 2:**
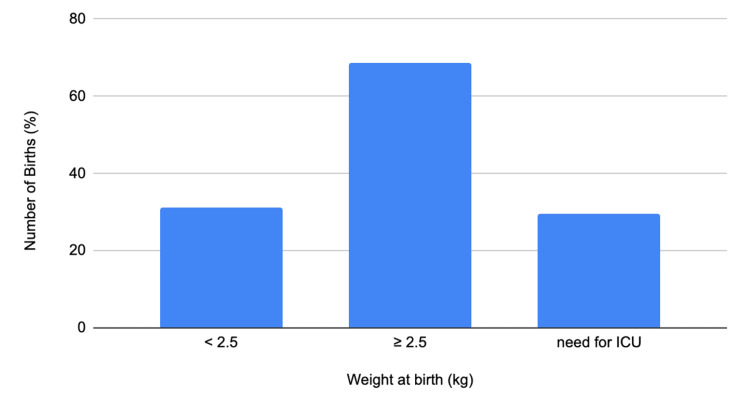
Fetal outcomes in a tertiary facility in south-western Nigeria between 2013 and 2015 by birth and weight Data from Management Outcome of Premature Rupture of Membranes in a Tertiary Health Facility in South Western Nigeria [14]. ICU = intensive care unit

In a retrospective study, the incidence of PROM in a southwestern tertiary facility in Nigeria was 4.1% with a perinatal mortality rate of 0.18 in 1000 births [14]. Nigeria was also identified as a large contributor to approximately half of the global figure for maternal and fetal deaths [15]. It was observed that 90% of PPROM and 9.8% of term PROM required neonatal intensive care. This study compared term PROM with PPROM, with findings suggestive of a high incidence of term PROM in their hospital. They also identified factors such as previous PROM, genital tract infections like bacterial vaginosis, cervical incompetence, overdistended uterus, and poor socioeconomic status and nutrition, which contribute to fetomaternal outcomes [14]. There was a higher incidence of PROM in multiparous and older women suspected to be due to varying degrees of cervical trauma from previous deliveries thereby affecting cervical competency. It was also noted that the GA at delivery following PPROM had a significant influence on the infant’s birth weight. Management strategies aim to reduce the rates of cesarean sections and operational vaginal deliveries [14]. Whereas conservative management led to spontaneous delivery compared to those induced, without a simultaneous increase in surgery rates [14]. The limitations of this study included the small sample size and the level of care. This study represents possible risk factors for PPROM in underserved regions. Though the index patient is multiparous with a previous vaginal delivery, revealing a possibility of cervical incompetence from the trauma of the previous vaginal delivery, it was not an identified risk factor in the patient, due to no history of prior second-trimester pregnancy losses, or previous cervical trauma from her first delivery, hence no cervical screening for cervical incompetence. Figure 2 below illustrates fetal outcomes of PROM from this study over two years.

**Figure 3:**
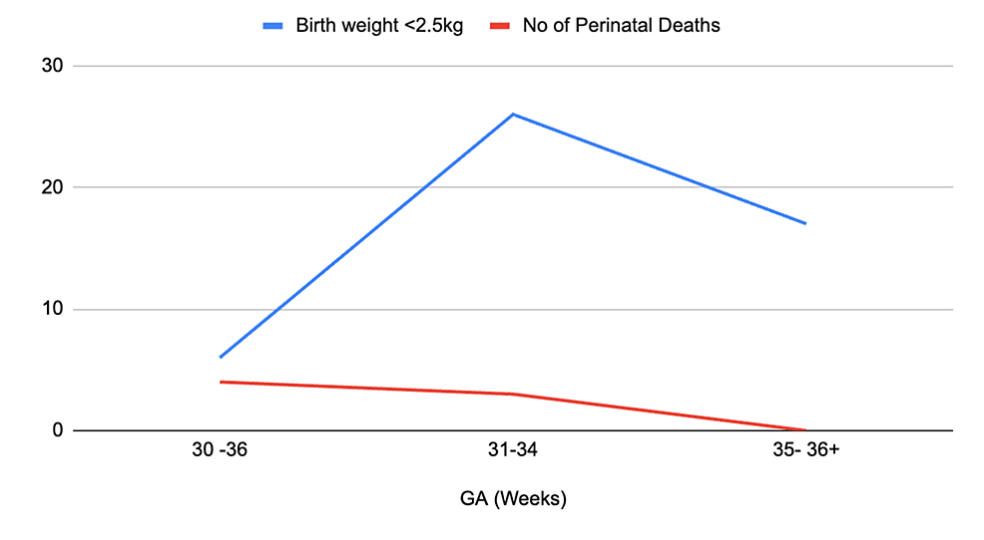
Trends in outcomes of PPROM at a south-eastern Nigerian hospital between 1999 and 2008 based on birth weight Data from The Incidence and Management Outcome of Preterm Premature Rupture of Membranes (PPROM) in a Tertiary Hospital in Nigeria [9] No = number GA = gestational age

Another Nigerian study in the southeastern region, showed a higher prevalence in women about 155 cm tall as opposed to shorter stature [9]. Infection was noted to be the most significant complication of PPROM up to 13.9% both in intra- and post-partum, with significant maternal morbidity of about 20% and perinatal mortality of 8.9%, despite liberal prophylactic antibiotics use. Shared decision-making and informed consent were crucial in successfully treating PPROM [9]. Small sample sizes and the geographical area are major limitations in this study in representing PPROM in Nigeria. The chances of extra uterine survival of preterm neonates less than 28 weeks in developing countries like Nigeria are very slim [14]. Thus, PPROM before 34 weeks of gestational age is managed conservatively with steroids, antibiotics, and close fetal monitoring to improve outcomes [9,14]. Figure 3 shows the trends in PPROM outcomes in this southeastern Nigerian tertiary facility over 10 years.

Comparison of current management guidelines

Many women with PPROM usually develop labor signs soon after membrane ruptures, however, there is a significant risk of ascending uterine infection in those who do not [16]. These infections are detrimental to both mother and fetus. Therefore, an accurate and prompt diagnosis is essential to detect PPROM, mitigate infection risks, and improve maternal perinatal outcomes [16]. About three-quarters of PPROM patients give birth within 24 hours, 9 out of 10 within 48 hours, and 19 out of 20 in 72 hours [12]. Whereas a relatively smaller proportion of PROM cases between 3% to 4% do not progress to labor within 168 hours (seven days) of ROM [12]. There is currently no gold standard to diagnose PPROM. When rupture and leakage of the amniotic fluid from the cervix is obvious, diagnosing PPROM is easier, however, if not observed, anamnesis and some tests can aid in diagnosis [7].

The American guideline advises against digital examination due to increased infection risk [17]. It however recommends speculum examination to visualize cervical leakage and vaginal pooling of amniotic fluid, a fern test of dried vaginal fluid, and PH testing of the fluid. PH between 7.1 and 7.3 indicates the presence of amniotic fluid on the speculum exam. Further supportive but unhelpful testing includes ultrasound for amniotic fluid volume and fetal fibronectin. Conclusive tests for PROM diagnosis include dye instillation by ultrasound guidance into the vagina or directly into maternal urine [17]. Management recommendations are expectant management or delivery after appropriate counseling, corticosteroids, antibiotics, and group B streptococcal (GBS) prophylaxis. However, shared decision-making and individualized care should be offered. Further, latency antibiotics should be avoided in known GBS status. Moreover, expectant management should not exceed 37+0/7 weeks and magnesium sulfate should not be administered prophylactically in PROM [17].

The National Institute for Health and Care Excellence's (NICE) guideline identified the use of placental alpha-microglobulin-1 and insulin-like growth factor binding protein-1 for correctly diagnosing PPROM. It also concluded that the presence of amniotic fluid pooling is an imminent sign of PPROM [16]. Thus, it was recommended that further diagnostic testing is unnecessary in the presence of amniotic fluid pooling, except for uncertainty in diagnosing PPROM. Despite the usefulness of these diagnostic tests, their cost-effectiveness is yet to be established [16]. The low quality of evidence provided by these tests invalidates their use alone in determining the level of care offered to women with PPROM [16]. Thus, a vaginal speculum exam to look for amniotic fluid pooling will determine the necessity of a diagnostic test. The clinical condition, medical and pregnancy history of the patient, and her gestational age are critical factors to guide evaluation and management. Nitrazine should not be used to diagnose PPROM, and no diagnostic tests should be performed in established labor following PPROM. Prescribing prophylactic antibiotics is discouraged if diagnostic tests are negative [16]. The Royal College guideline supplements the National Institute for Health and Care Excellence (NICE) guideline in offering corticosteroids at GA between 24+0/7 and 34+6/7 in imminent preterm delivery, as well as in women with increased risk of PPROM [18].

The Canadian guidelines recommend treating PPROM with two doses of intramuscular betamethasone 12 mg given 24 hours apart to accelerate fetal lung maturation. This is in addition to intravenous antibiotics with ampicillin 2 g and erythromycin 500 mg, every six hours [15]. A history of PROM is determined to be an unmistakable predictor of PROM in subsequent pregnancies, however, no positive correlation between PROM and behavioral factors such as drug use and cigarette smoking. Further, there was no relationship between socio-demographic factors (education, income, and adequacy of prenatal care), PROM, medical factors from the current pregnancy like urinary tract infection, chorioamnionitis, chlamydial or gonorrheal infections, and lower respiratory infections, and between PROM and prior therapeutic abortions, fetal loss/miscarriage, or preterm births [12].

At a Romanian hospital, expectant management was the preferred standard of care as it improved neonatal outcomes [7]. Four doses of 6 mg dexamethasone were administered every 12 hours to aid lung maturation and reduce the risk of other PPROM complications. The conditions for expectant management included gestational age 24 + 0/7 and 36 + 6/7 without complications such as chorioamnionitis, abruptio placenta, fatal death, fatal non-reassuring parameters, or advanced labor [7]. Higher latency periods have been associated with a higher incidence of infection, therefore medical care plays a vital role during the latency period, as there is no standard protocol [7]. Combined tocolytics and large-spectrum antibiotics were used to manage PPROM, with constant fetal monitoring.

In China, the most important risk factors for PROM in preterm neonates were smaller gestational age and expectant management [2]. For neonates born to mothers with PROM, the main interventions for PROM were induction of labor (IOL), the use of antibiotics, and expectant management. IOL according to the guidelines was protective for neonatal infectious diseases and early-onset pneumonia in term neonates while the influence of antibiotic use was insignificant. In preterm neonates, expectant management was still a risk factor for neonatal infectious diseases and early-onset pneumonia even when adjusted for gestational age [2]. Moreover, little is known about the epidemiology of PROM in China, and most data were obtained from American and European countries. In addition, the Chinese bulletin uses PROM guidelines from European and American countries for the White, Black, and Hispanic people [2]. Though there is much known about the epidemiology of PPROM and PROM in Nigeria, the Chinese population faces a similar challenge of not having a national guideline specific to their population.

Management of patients with PROM is determined by gestational age [3]. In Nigeria, the chances of survival before 28 completed weeks (age of viability) are very slim [14]. There is no national guideline for managing PPROM, however, admission for conservative or expectant management is the mainstay of care, as it reduces the chances of prematurity [9]. This involves antibiotics to prevent and/or treat intrauterine infections, steroid therapy for lung maturity, tocolysis to prevent uterine contractions (e.g. salbutamol as used in this patient), strict bed rest, and continuous fetal monitoring and surveillance [9,14]. However, the management of PPROM requires informed consent, discussion of the benefits and risks of treatment options, and shared decision-making with the patients [9]. Cervical ripening and IOL may be done in some cases, however, it was not possible in this patient, due to the prematurity, and fetal transverse lie which is a contraindication to cervical ripening. Timely and accurate diagnosis and the decision to allow spontaneous commencement of labor or to induce labor is a huge challenge in expectant management [14]. Surgical intervention may be indicated in some cases as in the index case.

Due to the financial constraints and lack of universal health insurance in under-resourced communities, such as that of the index case, there is non-affordability of serial ultrasound monitoring of the fetus before cesarean delivery. Ethical considerations, such as patients' knowledge and/or awareness of their diagnosis and management plan, as well as informed consent for treatment play vital roles in these settings [9]. The challenges of managing PPROM in this population are enormous. Factors such as resource-poor nations, lack of access to health care and qualified professionals, and late presentations contribute to the challenging management of PPROM in these communities. The demand for healthcare services in underserved areas is usually beyond the typical healthcare distribution of services in these populations [19]. Patients’ socioeconomic status can affect clinical decisions and thus can produce variability in care while trying to balance the current situation with the expected standard of care [19]. There is also variable availability of neonatal resuscitation facilities and specialist care across centers in Nigeria, thus, periviable fetuses with extremely low GA, for instance, would pose marked management challenges as they usually present with severe neonatal morbidities [5].

Recommendation

There is a pressing need to expand the research focus on PPROM and design a clear national protocol for the management of PPROM in Nigeria. While individualized treatment is encouraged, it is also important to develop a unifying global guideline for PPROM management such as through the World Health Organization (WHO). Advocacy of financial sponsorship of PPROM screening in developing nations of Africa will be beneficial to improve neonatal and maternal outcomes while maintaining patient confidentiality.

Study design and limitations

This is a single-center study of one intrinsic case presentation in South-South Nigeria, and thus may not be fully representative of the region. A multi-center study across the region is needed to substantiate this report. Gestational age was estimated largely from LMP and clinical examination at the first antenatal visit. Though the study included a one-year outcome of the survival of this baby, which has been uncomplicated, it opens up an area of research into the long-term outcomes of survivors of PPROM, especially in Nigeria. This case presentation is a pointer toward more research on the management and outcomes of PPROM in this region and Nigeria as a whole.

## Conclusions

A lack of updated and easily accessible national guidelines by the Society of Gynaecology & Obstetrics of Nigeria (SOGON) and a global guideline by the WHO was a significant contributor to the challenges in managing this patient. The available NICE guidelines provide variations that meet the needs of the population they serve in those regions. There is an apparent gap in clear guidelines for underserved communities and in resource-poor settings. In the index patient, there is a lack of availability of national guidelines, and patient care did not follow the NICE guidelines due to insufficient resources as per the standard required by NICE to make a prompt diagnosis and to provide appropriate management. For instance, diagnosing PPROM with placenta alpha-microglobulin-1 and insulin-like growth factor binding protein-1 is not routinely done in such settings. This causes clinicians to rely on clinical acumen, based on history and physical findings, such as the presence of amniotic pooling on speculum examination and amniotic fluid PH strip testing where available, to make a diagnosis and provide treatment. This is partially in keeping with the NICE guideline. It was also challenging to identify a risk factor, as the index case was a spontaneous PPROM. However, the administration of steroids and prophylactic antibiotics to this patient proved to have positively impacted both maternal and neonatal outcomes, especially fetal lung maturity before surgical intervention.
